# Endothelial Progenitor Cells for Diagnosis and Prognosis in Cardiovascular Disease

**DOI:** 10.1155/2016/8043792

**Published:** 2015-12-29

**Authors:** Caterina Oriana Aragona, Egidio Imbalzano, Federica Mamone, Valentina Cairo, Alberto Lo Gullo, Angela D'Ascola, Maria Adriana Sardo, Michele Scuruchi, Giorgio Basile, Antonino Saitta, Giuseppe Mandraffino

**Affiliations:** ^1^Department of Clinical and Experimental Medicine, University of Messina, Via Consolare Valeria, Gazzi, 98125 Messina, Italy; ^2^Department of Biochemical, Physiological and Nutritional Sciences, University of Messina, Via Consolare Valeria, Gazzi, 98125 Messina, Italy

## Abstract

*Objective*. To identify, evaluate, and synthesize evidence on the predictive power of circulating endothelial progenitor cells (EPCs) in cardiovascular disease, through a systematic review of quantitative studies. *Data Sources*. MEDLINE was searched using keywords related to “endothelial progenitor cells” and “endothelium” and, for the different categories, respectively, “smoking”; “blood pressure”; “diabetes mellitus” or “insulin resistance”; “dyslipidemia”; “aging” or “elderly”; “angina pectoris” or “myocardial infarction”; “stroke” or “cerebrovascular disease”; “homocysteine”; “C-reactive protein”; “vitamin D”. *Study Selection*. Database hits were evaluated against explicit inclusion criteria. From 927 database hits, 43 quantitative studies were included. *Data Syntheses*. EPC count has been suggested for cardiovascular risk estimation in the clinical practice, since it is currently accepted that EPCs can work as proangiogenic support cells, maintaining their importance as regenerative/reparative potential, and also as prognostic markers. *Conclusions*. EPCs showed an important role in identifying cardiovascular risk conditions, and to suggest their evaluation as predictor of outcomes appears to be reasonable in different defined clinical settings. Due to their capability of proliferation, circulation, and the development of functional progeny, great interest has been directed to therapeutic use of progenitor cells in atherosclerotic diseases. This trial is registered with registration number: Prospero CRD42015023717.

## 1. Introduction

Endothelial progenitors cells (EPCs) are a heterogeneous population of cells in different states of maturation, originated from bone marrow (BM). Since their identification by Asahara et al. [1], a great effort has been directed to explore the regenerative/reparative potential of EPCs, such as the capability of self-renewal, of starting reparative mechanisms, and of neoangiogenesis [2–4]. However, EPC isolation and characterization are still debated; in literature, two different approaches have been used to evaluate EPCs: identification of subpopulations based on surface markers from fresh blood and culture/colony assays. The methods for isolating circulating cells include adherence culture of total mononuclear cells obtained from fresh blood by density gradient centrifugation, positive preselection of mononuclear cells by antibodies against surface marker, and finally acquisition and analysis by flow cytometry [1, 5, 6]. Different culture methods were made by different working groups, which differ between them for the time of growth, for the media used, and for cell phenotypes. The common methods used may be summarized as EPC culture assay [7, 8], colony-forming unit-endothelial cell (CFU-EC) colony assay [9], and endothelial colony-forming cells (ECFC) [10–12]. Moreover, there is no clear evidence as to the existence of such culture-derived cells in vivo, and, more importantly, the relevance of such cells has not been functionally demonstrated in the clinical context [13, 14]. Many different surface antigens, often coexpressed by endothelial and hematopoietic cells, have been proposed and used to identify putative EPCs, including CD34, CD117, CD133, CD105, CD144, CD184, CD309 (KDR or VEGFR2), acetylated low-density lipoprotein, and various plant lectins [15], so the question of which cell phenotype better identifies the “true” circulating EPC remains unsolved; the more widely studied progenitor phenotypes, also despite some evidence in clinical studies, do not give rise to mature endothelial cells in cultures and are different from endothelial colony-forming cells [12, 16, 17], although the ability to differentiate in vivo into a broad range of cell types of different organs and systems, including cardiomyocytes, smooth muscle cells, and endothelial progenitor cells (EPCs), as well as hematopoietic, stromal, and epithelial cells, has been suggested, with a role in cooperating with EPCs for postnatal vasculogenesis, working as proangiogenic support cells, participating in the turnover of healthy and damaged endothelium, maintaining their importance as regenerative/reparative potential, and also as prognostic markers [15–22], likely delaying the development of atherosclerosis and cardiovascular disease (CVD). Moreover, it has been suggested that different circulating progenitors show an important differentiation and also transdifferentiation ability [22–24].

Over the last 15 years, many studies focused on the role of EPCs in clinical conditions characterized by increased cardiovascular (CV) risk, such as smoke exposure, hypertension, diabetes, dyslipidemia, and aging, and in general by atherosclerotic disease (coronary artery disease (CAD), acute myocardial infarction (AMI), cerebrovascular disease, and stroke).

This literature review aims to give an overview on the current stand of knowledge on the so-called EPCs, including insights into their use for diagnosis and prognosis of CVD.

## 2. Methods

This review was performed following methods that are reported in the PRISMA Statement. A systematic literature search was conducted in PubMed MEDLINE from January 2000 to December 2014. MEDLINE was searched using keywords related to “endothelial progenitor cells” and “endothelium” and, for the different categories, respectively, “smoking”; “blood pressure”; “diabetes mellitus” or “insulin resistance”; “dyslipidemia”; “aging” or “elderly”; “angina pectoris” or “myocardial infarction”; “stroke” or “cerebrovascular disease”; “homocysteine”; “C-reactive protein”; “vitamin D”. We identified 299 out of 927 publications, so divided: 32 out of 47 for smoking; 21 out 78 for blood pressure; 63 out of 137 for diabetes mellitus/insulin resistance; 13 out of 32 for dyslipidemia; 24 out of 431 for aging or elderly; 86 out of 101 for angina pectoris/myocardial infarction; 45 out of 86 for stroke and cerebrovascular disease; 15 out of 15 for “non-classic” risk factors. To determine study eligibility, two authors worked separately, in two different stages: in the first stage the titles and abstracts of all search results were screened by an author using predefined keywords. In the second stage another author provided determining articles eligibility. In vivo, ex vivo, and in vitro studies with only cellular or molecular endpoints were excluded. Case reports, narrative reviews, and non-English publications were also excluded. Subsequent choices have been performed according to the number of patients, the type of study population, and the type of population of comparison, subset of cells by surface antigen. We would clarify that although the research by keywords “elderly” and “aging” found 431 articles, many of these were not eligible for the review; we have excluded articles with population aged less than 60 years, non-CV associated morbidities (e.g., carcinoma, chronic lung obstructive disease, and autoimmune diseases), and interventional study. The major limitation in this keywords-based research is due to the ambiguity of “aging/senescence” key; in literature, in fact, these terms are currently used for both cellular and population aging/senescence; consistently, we had to exclude a great number of studies, and only 6 articles were included in the revision.

## 3. Results

**Figure 1:**
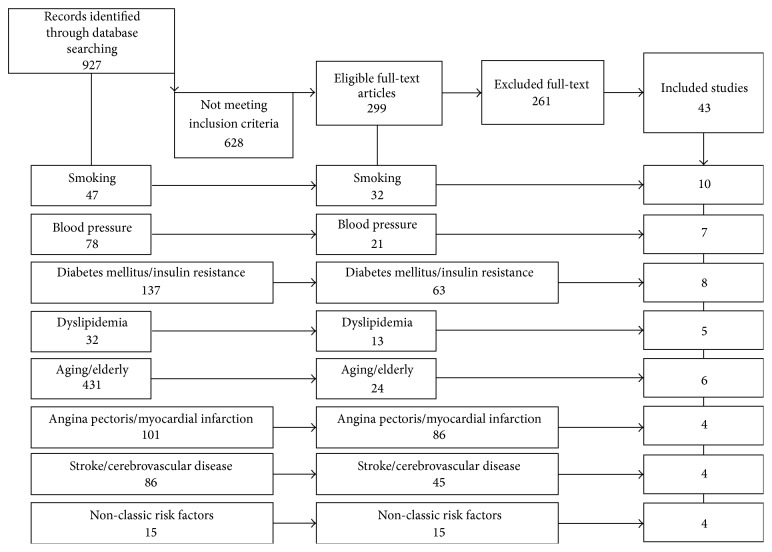
Flow diagram for patients exclusion.

The flow diagram for patients inclusion/exclusion is reported in Figure 1. At last, the included studies were 43 and we can divide them as follows: 10 for smoking, 7 for hypertension, 8 for diabetes mellitus and insulin resistance, 5 for dyslipidemia, 6 for aging, 4 for angina and myocardial infarction, 4 for stroke and cerebrovascular disease, and 4 for “non-classic” risk factors. Three articles have been revised into more different categories (smoking and hypertension [8]; smoking, hypertension, and dyslipidemia [9, 25]).

### 3.1. Cigarette Smoking and EPCs

Cigarette smoking (CS) is an important risk factor for CVD and has been reported to contain numerous toxic compounds which increases reactive oxygen species (ROS) production in vivo and oxidative stress [26]. Excessive ROS production/accumulation can shorten cellular lifespan and facilitate the development of CV lesions [27]. CS may induce various pathological alterations causing CV lesions. Indeed, CS reduces the synthesis of nitric oxide (NO), represses endothelium function and dilation, and induces an inflammatory response through the production of several mediators [28–30]. In the last few years, several authors have focused on the relationship between CS and EPCs (Table 1). In 2001, Vasa et al. suggested that EPC count in smokers was correlated with total number of risk factors and the analysis of the individual risk factors indicated that smoking is an important determinant of the numbers of circulating EPCs [8]. Hill et al. evaluated EPC numbers and activity in relation to CV risk factors [9]. Multivariate regression analysis was performed to determine whether the number of EPCs was associated with age, race, body-mass index, CS, hypertension, diabetes, total cholesterol, or glucose levels: they found no correlation between EPCs and individual risk factors and, conversely, found a strong correlation between the number of EPCs and the subjects' combined Framingham risk factor score [9]. Yue et al. found that circulating levels of EPC were significantly lower in smokers with CAD compared to controls and nonsmokers with CAD [31]. In patients with CAD, Werner et al. reported, by univariate analyses, that smoking was associated with high baseline levels of EPCs [25]. Similarly, Mobarrez et al. evaluated EPC levels in healthy intermittent/sporadic smokers; they found that CD34+ was significantly increased after smoking one cigarette [32]. Other authors have shown that the number of EPCs is directly proportional to the number of cigarettes smoked; moreover, the amount of EPCs presents rapid recovery after smoking cessation and then, after the resumption of smoking, it again falls to the levels similar to that before quitting smoking. At last, this recovery was greater in light smokers compared to heavy smokers [33]. It has been proposed that smoking may change the BM setting, decreasing the EPCs mobilization from the BM, probably through the inhibition of NO release. Similarly, Michaud et al. found that the number of EPCs was lower in smokers (reduced by more than 50% compared to control). In addition, they have found that ROS formation was significantly increased in EPCs isolated from smokers. The potential mechanisms responsible for the negative effect of smoking on EPCs were suggested to include increased oxidative stress, decreased NO availability, and impaired EPC differentiation towards an endothelial phenotype [34]. EPCs express constitutionally high levels of antioxidative enzymes, including glutathione peroxidase (GPx-1), manganese superoxide dismutase (MnSOD), and catalase (CAT), which are able to limit the damage of oxidative stress by reducing intracellular ROS concentration [35–37]. To investigate the relationships between CS, number of EPCs, and intracellular levels of ROS, Mandraffino et al. reported that smokers presented with higher intracellular MnSOD expression and activity, which were positively correlated with ROS, and also that the inducible CAT and GPx-1 enzymes were underexpressed in smokers as compared to nonsmoker controls. In this model it could be observed that the redox homeostasis appeared to be imbalanced and the ROS formation rate to exceed the capacity of the antioxidative defense system in circulating cells isolated from smokers. They suggest that the chronic inflammatory stress induced by smoke exposure may affect the system of antioxidant enzymes and that the impairment of this enzymatic balance may result in a reduction of EPCs [38]. A few years ago a trial remarked the role of cigarette smoking in patients with myocardial infarction; in the BONAMI trial, indeed, authors found that in patients with AMI active smoking impairs myocardial viability recovery [39] and in a subsequent work showed that smoking subjects with myocardial infarction, as compared to nonsmoking and former smoking patients, had an increased BM cells count. In addition, the number of circulating EPCs in nonsmokers and former smokers was predictive of reducing infarction area measured by cardiac single photon emission computed tomography at three months after infarction. Furthermore they confirmed that in smokers circulating EPC levels were lower and their migration was impaired. These data could assume that smoking-related EPC alterations participate in the impairment of cardiac function recovery observed in smokers [40].

### 3.2. Hypertension and EPCs

Several studies have shown that the blood pressure values have a close relationship with the incidence of CVD, such as stroke, CAD, sudden death, heart failure, and peripheral arterial disease [41, 42]. On the basis of epidemiological and pathophysiological significance of hypertension, many authors have focused on the relationship between this condition and EPCs, but with conflicting results (Table 2). Vasa et al. showed an impaired EPC function in CAD patients: the evaluation of the individual risk factors revealed that EPC migration was inhibited in patients with hypertension and the result remains the same even with multivariate analysis [8]. Hill et al. found a strong correlation between the number of circulating EPCs and the patient's combined Framingham risk factor score; moreover, they saw also a correlation between a reduced number of CFU-ECs and hypertension, which however disappeared after adjusting for age [9]. Similarly, Werner et al. observed that the correlation between low EPC count and high blood pressure values disappears by the multivariate analysis [25]. Oliveras et al. investigated the number of circulating cells in patients with refractory hypertension (RHT), and they interestingly reported that the concentration of EPCs was significantly reduced in RHT as compared to healthy subjects [43]; consistently, preliminary data about renal denervation in RHT patients showed that after the procedure the improvement of blood pressure control was accompanied by the increase of peripheral CD34+ cell number [44]. MacEneaney et al. evaluated EPCs in prehypertensive adults: in particular, in subjects with systolic blood pressure greater than 130 mmHg but lower than 139 mmHg, the ability of EPCs to form colonies is impaired compared to normotensive subjects [45]. Otherwise, Delva et al., by studying the number and functional activity of EPCs in essential hypertensive patients, observed that the EPC number was not statistically different from that found in control subjects [46]. Mandraffino et al. divided their hypertensive patients into two groups according to the presence of isolated arterial stiffening (AS) or AS and both carotid intima-media thickening and left ventricular hypertrophy; they found that hypertensives with more advanced vascular and cardiac involvement had fewer circulating CD34+ cells than hypertensives with earlier vascular lesions but more than normotensive controls [47]; moreover, they suggested that different EPCs phenotypes may behave differently in different subsets of hypertensive patients.

The discrepancies reported in all these works could be due to differences in study design (evaluation of EPC phenotypes, count method, activity or function, and cell culture), population inclusion criteria, and CV risk or organ damage associated. We should consider that there is no unambiguous consensus about what techniques and methods should be used to identify EPCs [48, 49]. Different cell phenotypes have been used by different authors; accordingly, a straightforward comparison among the studies remains difficult to be performed. However, EPC amount in hypertensive patients could be influenced by concomitant treatments or comorbidities that could influence their BM release or their capacity to resist to oxidative stress or to respond to proapoptotic stimuli.

### 3.3. Diabetes and EPCs

The mechanisms of endothelial damage induced by hyperglycemia are well known and it is also known that endothelial damage over time may cause both micro- and macroangiopathic complications [50]. Some authors tried to correlate endothelial damage to EPCs in diabetic patients (Table 3). In 20 patients affected by type 1 diabetes (T1DM) compared with 20 age- and sex-matched control subjects, Loomans et al. found that the number of cultured EPCs was reduced and was inversely related with levels of glycated hemoglobin (HbA1c) [51]. Similarly, Kusuyama et al. before (2006) [52] and Egan et al. later (2008) [53] showed that in patients affected by type 2 diabetes mellitus (T2DM) EPC count is reduced with respect to healthy controls and significantly related to HbA1c levels. Moreover, it has been already reported that the number of putative EPCs was lower as more numerous were the complications [53]. The reduction of the circulating CD34+ progenitor cells in early stages of T2DM can be suggested in individuals with impaired glucose tolerance; this reduction persists over time and worsens in patients with advanced complications [54]. Lombardo et al. found low EPC levels in their diabetic population, both with and without vascular complications, and propose an altered process of maturation/commitment of EPCs rather than a failure of their production/mobilization from the BM to explain endothelial dysfunction [55]. On the other hand, several authors have shown increased circulating and cultured EPCs in patients with advanced or proliferative retinopathy, both in T1DM [56] and in T2DM [57]. In detail, Asnaghi et al. found that EPC count is increased in patients with proliferative retinopathy compared to diabetics without retinopathy, and the number of EPCs in healthy controls is significantly higher also than in patients without retinopathy [56]; in this setting, cell proliferation/mobilization could be explained by the stimulus to the retinal angiogenesis. In addition to increased glucose levels, T2DM is also characterized by a condition known as insulin resistance (IR), which is defined as a reduction of sensitivity and/or reactivity of the target cells to plasmatic insulin [58]. Insulin-mediated endothelial damage has been proposed, and it has been shown that a treatment with insulin-sensitizing drugs (PPAR-*γ* agonist; metformin) restores EPCs number in T2DM [59, 60], independently of glycemic control [60]. The IR effects on EPCs might be due, at least indirectly, to the systemic activities of insulin (e.g., oxidative stress, inflammation, and increased free fatty acids) or directly to BM cells and EPCs [61]. More studies are needed to clarify the complex interplay between IR, EPCs, endothelial damage, and repair.

### 3.4. Dyslipidemia and EPCs

It is known that the process of atherosclerosis is, at least in part, determined by a progressive accumulation of lipids within the vascular wall. Numerous experimental and epidemiological studies showed a causal relationship between hyperlipidemia and/or high levels of low-density lipoprotein cholesterol (LDL-C) and atherosclerosis [62, 63]. Starting from the paradigm of Ross that atherosclerosis is an inflammatory disease of the vascular endothelium [64], already in 2003 Hill et al. found, in relatively healthy subjects with different cardiovascular risk factors, that the number of CFU-ECs was significantly reduced, but after adjusting for age and the individual risk factors (cholesterol levels, hypertension, and diabetes), only hypercholesterolemia remained to be significant [9]. Similarly, Chen et al. observed that EPC number was significantly lower in patients with hypercholesterolemia with respect to control subjects and that it was inversely correlated with total cholesterol and LDL-C levels [65]. Similar results were obtained in vitro: the exposure of cultured EPC to oxidized LDL induces a dose-dependent impairment of their activity, accelerates the rate of cell senescence (possibly by telomerase inactivation), and could be associated with a significant reduction in EPC numbers in vivo [66] (Table 4). Already in the Framingham study, low high-density lipoprotein cholesterol (HDL-C) levels have been associated with high incidence of CVD [67], and also HDL-C levels were associated with EPC count. In detail, circulating EPCs decrease was found in hypercholesterolemic patients, and the reduction appeared to be more evident in the low HDL-C subgroup [68]. Werner et al. found that low EPCs levels were associated with increased LDL-C levels and that therapy with statins was associated with higher cell count [25]. The statin effects on EPC activity appear to be independent of the impact on LDL-C reduction, as shown by the comparison of simvastatin with ezetimibe administration [69], suggesting that the beneficial effect of lipid lowering drugs on the endothelium health status may be enhanced by EPC stimulation.

### 3.5. Aging and EPCs

Aging is one of the main risk factors for the development of CVD, because several changes occur in the structure of organs and systems with age, such as complex alterations in the vasculature [70–72]. Tissue repair ability may not be indefinite; it has been proposed that once the capacity is exhausted, a chronic inflammatory process leads to evident pathological manifestations [73]. To explain how age may affect EPCs survival, Dimmeler and Vasa-Nicotera proposed an increased turnover rate with increased susceptibility to apoptosis; they suggested in progenitor cells an imbalance in pro- and antiapoptotic factors, a decline in the antioxidant defense, or telomere shortening and dysfunction [74]. Heiss et al. enrolled 20 young and 20 old healthy subjects without clinical evidence of other CV risk factors and found no quantitative difference in EPCs. They described that culture-enriched EPCs from elderly were impaired in terms of proliferation, migration, and survival [75]. Other authors reported an inverse relation between age and EPC count: a reduction in the number of circulating EPCs was seen in elderly as compared with younger adults [76]. Interestingly, Xia et al. found in elderly healthy men lower levels of EPCs compared to young healthy controls. Furthermore, transplantation of EPCs from young people but not EPCs from the elderly markedly accelerated reendothelialization of the injured arteries in a model. Authors propose that shear stress exerts beneficial effects on human EPCs for endothelial protection [77]. The question of whether the cells may correlate with people's survival has been also raised; Mandraffino et al. designed a study to evaluate the ability of CD34+ progenitor cells to predict long-term survival in a population of octogenarians. They reported, after 7 years of follow-up, a higher incidence of deaths in patients with lower baseline levels of circulating CD34+ cells. Moreover, the CD34+ cell number recorded at enrolment was significantly higher in subjects who reached older age at death or were still living at the end of observation period, with respect to the subjects who died; in detail, most of subjects who died had lower CD34+ cell number (1st tertile), whereas most of still living people had higher CD34+ cells (3rd tertile). They suggested that the higher the CD34+ cell number at baseline, the greater the chances of reaching an older age; the association between CD34+ cell count and longevity was maintained also after adjustment for classic CV risk factors [78]. Also lifestyle and diet have been suggested as potential modulators of EPC amount and function in old people [79, 80] (Table 5).

### 3.6. CVD and EPCs

Since their discovery [1], endothelial progenitors have attracted considerable interest because of their association with the development of CVD. The possibility to explain, at least in part, the mechanisms underlying the endothelial damage and the opportunity to identify a group of circulating cells with the ability to recover this damage have given further input to the research in this area. Therefore, after discussing the individual CV risk factors, we can summarize, in part, what is reported in the literature about EPCs in patients with major CV risk (Table 6). Heeschen et al. isolated hematopoietic progenitor cells from BM aspirates in 18 patients with chronic ischemic cardiomyopathy and 8 healthy control subjects. They did not observe differences in the number of progenitor cells in the BM, but, in vitro, the functional capacity of EPCs (evaluated as colony-forming activity and migratory response) appeared significantly reduced in chronic ischemic patients compared to controls [81]. George et al. selected patients with unstable and stable angina; patients with unstable angina had significantly greater numbers of circulating EPCs and EPC-CFUs [82]. After acute myocardial infarction, Shintani et al. reported that CD34+ cells number did not differ between the infarction patients and controls on day 1, but cells levels appeared to linearly grow in the days after the event, reaching a peak after 7 days [83]. Massa et al. found that the percentage of total circulating CD34+ cells was significantly higher in patients with myocardial infarction at admission than controls. The longitudinal study of patients with myocardial infarction showed a decreasing trend of the number of circulating CD34+ cells, which at day 7 was statistically lower than at admission, although it was higher than that of controls and became comparable to that of controls within 60 days [84]. Ghani et al. reported that the number of EPCs was significantly lower in patients with cerebrovascular disease (acute or chronic) than in control subjects and was not lower in patients with acute stroke compared with patients with history of cerebral ischemic events [85]. In patients with a history of cerebral ischemic events, Taguchi et al. found no association between EPCs and the degree of cerebrovascular atherosclerosis; conversely, after acute cerebral infarction, the EPCs gradually were increasing, returning to baseline levels after 30 days [86]. Yip et al. found levels of circulating EPCs higher in ischemic stroke patients with respect to control subjects. Moreover, patients with recurrent ischemic stroke had lower levels of circulating EPCs than patients with a first event. Impaired EPCs levels during the acute phase were associated with absence of major adverse clinical outcomes. The authors suggested that patients with low EPC count have a reduced capacity for angiogenesis, repair of endothelial damage, and formation of collateral vessels [87]. Similarly, other researchers reported a better prognosis related to the higher EPC count during the ischemic event [88].

### 3.7. Residual CV Risk and EPCs

In the last few years increasing attention was dedicated to estimate the association between EPCs and the “non-classic” risk factors. Circulating high-sensitivity C-reactive protein (CRP) represents a potential independent predictor of vascular damage [89, 90]. Initially proposed as a biomarker, CRP was subsequently suggested as a player in atherogenesis [91], although its role has not been definitely disclosed [92]. Verma et al. demonstrated that EPCs incubated with human recombinant CRP, at concentrations known to predict adverse vascular outcomes, exhibited decreased survival, promoting apoptosis; the reduction of EPCs appears CRP dose-dependent [93]. Homocysteine (Hcy), another emergent CV risk factor [94], was shown to decrease EPC count and to impair EPCs activity [95]. Interestingly, in patients with newly diagnosed hypertension, Bogdanski et al. found that Hcy levels are significantly associated with increased carotid IMT and decreased number of CFU-ECs and proposed as explanation that Hcy may interfere with the redox setting [96]. Vitamin D deficiency has been associated with CVD [97, 98]; more recently, vitamin D has been suggested to exert effects on the CV system [99]. Mikirova et al. found that vitamin D status has an effect on EPC number and on the ability of peripheral mononuclear cells to differentiate in angiogenic cells. In their study, mean values of EPCs for subjects with a sufficient level of vitamin D were higher than for subjects with an insufficient or deficient level of vitamin D. They suggest that a higher plasmatic level of vitamin D may have an impact on the ability of stem cells in circulation to differentiate in endothelial phenotype [100]. However, further studies are needed to explain the possible correlation between these “non-classic” CV risk factors and the EPCs.

### 3.8. Therapeutic Purposes

In 2001 the Transplantation of Progenitor Cells and Regeneration Enhancement in Acute Myocardial Infarction (TOPCARE-AMI) study was started. Assmus et al. demonstrated, in a cohort of patients with AMI treated by coronary stenting for reperfusion, that the intracoronary infusion of EPCs was associated with a significant increase in left ventricular (LV) ejection fraction, a deep gain in wall motion abnormalities in the infarct area, and a significant reduction in end-systolic LV volumes 4 months after the AMI, suggesting a beneficial effect on postinfarction remodeling processes [101]. Fifty-five patients completed the five-year follow-up: data show a persistent improvement of LV ejection fraction and a reduction in functional infarct size [102]. Strauer et al. found similar results after 3 months of follow-up. They reported a reduction of infarct region and an improvement in wall movement velocity, in LV end-systolic volume and contractility, and in myocardial perfusion [103]. In another randomized study, Janssens et al. observed that intracoronary transfer of autologous BM cells does not augment recovery of global LV function after AMI but could favorably affect infarct remodeling [104]. Other encouraging results derive from the Reinfusion of Enriched Progenitor Cells and Infarct Remodeling in Acute Myocardial Infarction (REPAIR-AMI) trial, in which LV angiography was used to assess LV ejection fraction before and 4 months after BM cells delivery: patients with more severely depressed systolic function at baseline obtain the greatest benefit from BM cells therapy [105]. In the BOOST trial, intracoronary autologous BM cells transfer provides a sustained overall treatment effect on echocardiographic parameters of diastolic function in patients after AMI [106]. However, this effect declines over long-term follow-up of 5 years and is basically related to an early improvement of parameters of diastolic function at 6 and 18 months [107]. It is important to note that in all these trials the safety and the reproducibility of the reinfusion therapy have been underlined. More recently, another method that exploits EPCs in coronary disease was made, the EPC capture stent (ECS). The stent struts are coated with a biocompatible matrix with antihuman CD34+ antibodies covalently attached. After the EPCs are immobilized on the stent surface, these cells rapidly differentiate into a functional endothelial layer. This technology was designed to inhibit stent-related thrombus formation and neointimal hyperplasia. The Healthy Endothelial Accelerated Lining Inhibits Neointimal Growth (HEALING) First-In-Man and HEALING II study have demonstrated safety and efficacy of ECS and a favorable clinical outcome after one year [108–110]. To evaluate clinical outcomes after stenting of coronary bifurcation lesions in a real-world population the e-HEALING registry was created: a multicenter, prospective, worldwide, postapproval registry that evaluated the ECS in 4996 patients. Data taken from the e-HEALING registry showed that coronary bifurcation stenting with the ECS resulted in favorable 12-month clinical outcomes and low incidences of repeat revascularizations and ST [111, 112].

Other fields of application of autologous BM cells transfer are peripheral arterial obstructive disease and critical limb ischemia. It is known that the gold-standard treatment of these peripheral atherosclerotic complications is surgical or endovascular revascularization. However, one-third of patients are not candidates for invasive interventions. Compagna et al. recently have reviewed the literature and have confirmed the beneficial role of cell therapy in reducing the rate of major amputations, improving distal perfusion, ankle-brachial index, and partial pressure of oxygen, increasing walking distance, and reducing pain [113]. These data are confirmed in a meta-analysis involving 1214 patients treated with BM stem cell-based therapy [114].

## 4. Conclusions

In recent years EPCs have taken an important role in scientific research for diagnosis and prognosis in CVD, although identification, characterization, and function in vascular biology of this circulating progenitor cell subset are still being debated [14]. Due to their ability to proliferate, circulate, and originate functional progeny, great interest has been directed also to therapeutic use of progenitor cells in atherosclerotic diseases.

It is clear that EPCs are involved in vascular rearrangement during endothelial insult or damage. In fact, after endothelial damage a mechanism of repair starts, and circulating progenitors contribute to this process. These circulating BM-derived cells comprise several different subsets of cell types, displaying different features, although interacting and sometimes overlapping with each other, to produce, maintain, and repair functional vessels [15, 21, 115, 116].

Multiple divergent types of circulating blood cell can display endothelial characteristics and have been referred to as “EPC” in the literature. Our literature revision reveals how, using keywords like “endothelial progenitor cells”, it is possible to find researches about different clusters of cells, identified by different antigenic markers and with the ability to differentiate into a broad spectrum of different cellular lines including cardiomyocytes, smooth muscle cells, and progenitor cells, as well as hematopoietic, stromal, and epithelial cells, with a role in cooperating with EPCs for postnatal vasculogenesis, working as proangiogenic support cells [15–22].

Importantly, there are some inconsistencies between studies, which are likely to be related to either differences in the methods of EPC characterization or patient selection. As EPC levels are influenced by several factors, it is important to further understand the mechanisms by which EPCs are affected at different stages of the different types of CVD. This knowledge will help to focus specific interventions aimed at enhancing EPC numbers and function in patients with atherosclerotic disease.

Indeed, different EPCs showed and shared the ability to identify CV risk conditions and to predict better/worse CV outcome, thus suggesting their evaluation as a reasonable marker in different defined clinical settings. Lastly, there are several promising studies to suggest EPCs as a novel therapy for CVD. A broad consensus appears to be needed about the definition of EPC, as well as about the other cell types cooperating in vivo with EPCs and working as support cells, but not EPCs.

New studies should be referred to the definition of standardized methods for the identification and use of EPCs as diagnostic, prognostic, and therapeutic indices in the common clinical practice.

## Figures and Tables

**Table 1:** Smoking and EPCs.

Study	Population	Effect on EPCs number
Vasa et al. [8]	CAD	↓ CD34+/KDR+
Hill et al. [9]	CVR	NE
Yue et al. [31]	CAD	↓ CD34/KDR+; ↓ CD133+/KDR+
Werner et al. [25]	CVD	↑ CD34+/KDR+; ↓ CFU-EC
Kondo et al. [33]	NACVR	↓ CD45 low/CD34+/CD133+/KDR+
Michaud et al. [34]	NACVR	↓ EPCs
Mandraffino et al. [38]	NACVR	↓ CD34+, CD133+, CD34+/CD133+, CD34+/KDR+, CD133+/KDR+
Mobarrez et al. [32]	Healthy	↓ CD34+/KDR+
Roncalli et al. [39]	AMI	↓ CD45+/CD34+/CD133+/KDR+
Lamirault et al. [40]	AMI	↓ EPCs

NE: no effect, CVR: cardiovascular risk, NACVR: no-additional CVR, CAD: coronary artery disease, CVD: cardiovascular disease, and AMI: acute myocardial infarction.

**Table 2:** Hypertension and EPCs.

Study	Population	Effect on EPCs number
Vasa et al. [8]	CAD	NE
Hill et al. [9]	CVR	↓ CFU-EC
Werner et al. [25]	CVD	↓ CD133+
MacEneaney et al. [45]	Prehypertensive	↓ EPCs, ↓ CFU-EC
Oliveras et al. [43]	RHT	↓ CD45+/CD34+/CD133+; ↓ CFU-EC
Delva et al. [46]	Hypertensive	NE cells number and CFU
Mandraffino et al. [47]	Hypertensive	↑ CD34+

NE: no effect, CAD: coronary artery disease, CVR: cardiovascular risk, CVD: cardiovascular disease, and RHT: refractory hypertension.

**Table 3:** Diabetes and EPCs.

Study	Population	Effect on EPCs number
Loomans et al. [51]	T1DM	↓ in culture
Kusuyama et al. [52]	T1DM	↓ in culture
Egan et al. [53]	T2DM	↓ EPCs
Lombardo et al. [55]	T2DM	↓ EPCs
Asnaghi et al. [56]	T1DM retinopathy	↑ EPCs
	T1DM no-retinopathy	↓ EPCs
Brunner et al. [57]	T2DM retinopathy without macrovascular complications	↓ EPCs
	T2DM retinopathy with macrovascular complications	↑ EPCs

T1DM: type 1 diabetes mellitus, T2DM: type 2 diabetes mellitus.

**Table 4:** Dyslipidemia and EPCs.

Study	Population	Effect on EPCs number
Hill et al. [9]	CVR	↓ CFU-EC
Chen et al. [65]	CAD	↓ in culture
Werner et al. [25]	CVD	↓ CD133+
Rossi et al. [68]	High cholesterol levels	↓ CD34+/CD133+

CAD: coronary artery disease, CVR: cardiovascular risk, and CVD: cardiovascular disease.

**Table 5:** Aging and EPCs.

Study	Population	Effect on EPCs number
Heiss et al. [75]	Healthy	↓ in culture
Jie et al. [76]	Healthy	↓ CD34+/KDR+
Xia et al. [77]	Healthy	↓ CD34+/KDR+
Mandraffino et al. [78]	CVR	↓ CD34+

CVR: cardiovascular risk.

**Table 6:** CVD and EPCs.

Study	Population	Effect on EPCs number
Heeschen et al. [81]	CAD versus healthy	NE
George et al. [82]	Unstable angina versus stable angina	↑ EPCs, ↑ CFU-ECs
Shintani et al. [83]	AMI versus stable angina	↑ CD34+, ↑ CFU-ECs
Massa et al. [84]	AMI versus healthy	↑ CD34+/KDR+
Ghani et al. [85]	Cerebral disease versus healthy	↓ CFU-ECs
Taguchi et al. [86]	Acute cerebral infarction	↓ CD34+/CD133+
Yip et al. [87]	Cerebral disease versus CVR	↑ EPCs

NE: no effect, CAD: coronary artery disease, AMI: acute myocardial infarction, and CVR: cardiovascular risk.

## Abbreviations

AMI: Acute myocardial infarction
AS: Arterial stiffening
BM: Bone marrow
CAD: Coronary artery disease
CAT: Catalase
ECFC: Endothelial colony-forming cells
CFU-EC: Colony-forming unit-endothelial cell
CRP: High-sensitivity C-reactive protein
CS: Cigarette smoking
CV: Cardiovascular
CVD: Cardiovascular disease
ECS: EPC capture stent
EPC: Endothelial progenitor cell
GPx-1: Glutathione peroxidase
HbA1c: Glycated hemoglobin
Hcy: Homocysteine
HDL-C: High-density lipoprotein cholesterol
IR: Insulin resistance
LDL-C: Low-density lipoprotein cholesterol
LV: Left ventricular
MnSOD: Manganese superoxide dismutase
NO: Nitric oxide
RHT: Refractory hypertension
ROS: Reactive oxygen species
T1DM: Type 1 diabetes mellitus
T2DM: Type 2 diabetes mellitus.
